# Therapeutic targeting of ALS pathways: Refocusing an incomplete picture

**DOI:** 10.1002/acn3.51887

**Published:** 2023-08-28

**Authors:** Nicholas J. Maragakis, Mamede de Carvalho, Michael D. Weiss

**Affiliations:** ^1^ Department of Neurology Johns Hopkins University Baltimore Maryland USA; ^2^ Faculdade de Medicina Insqatituto de Medicina Molecular João Lobo Antunes, Centro Académico de Medicina de Lisboa, Universidade de Lisboa Lisbon Portugal; ^3^ Department of Neurology University of Washington Seattle Washington USA

## Abstract

Numerous potential amyotrophic lateral sclerosis (ALS)‐relevant pathways have been hypothesized and studied preclinically, with subsequent translation to clinical trial. However, few successes have been observed with only modest effects. Along with an improved but incomplete understanding of ALS as a neurodegenerative disease is the evolution of more sophisticated and diverse in vitro and in vivo preclinical modeling platforms, as well as clinical trial designs. We highlight proposed pathological pathways that have been major therapeutic targets for investigational compounds. It is likely that the failures of so many of these therapeutic compounds may not have occurred because of lack of efficacy but rather because of a lack of preclinical modeling that would help define an appropriate disease pathway, as well as a failure to establish target engagement. These challenges are compounded by shortcomings in clinical trial design, including lack of biomarkers that could predict clinical success and studies that are underpowered. Although research investments have provided abundant insights into new ALS‐relevant pathways, most have not yet been developed more fully to result in clinical study. In this review, we detail some of the important, well‐established pathways, the therapeutics targeting them, and the subsequent clinical design. With an understanding of some of the shortcomings in translational efforts over the last three decades of ALS investigation, we propose that scientists and clinicians may choose to revisit some of these therapeutic pathways reviewed here with an eye toward improving preclinical modeling, biomarker development, and the investment in more sophisticated clinical trial designs.

## Introduction

Numerous therapeutic strategies for treating amyotrophic lateral sclerosis (ALS) have been studied in individuals with the disease. However, few successes have been recorded, and results in slowing disease progression have been modest. Our knowledge about the genetic and pathophysiological underpinnings of the disease has certainly grown in the last three decades, and it is clear that ALS is much more heterogeneous in its presentation and progression than was appreciated with the positive clinical trials testing riluzole for ALS during the 1990s, a drug with various other important mechanisms for neuroprotection in addition to its antiglutamatergic action.^1^, ^2^ With important discoveries regarding the genetic underpinnings of some patients with ALS, it is now well accepted that numerous biological pathways are involved in disease onset and progression. These discoveries require careful thought regarding therapeutic applications and decisions about how ALS therapeutics will be targeted to specific, but largely undefined, subsets of the disease.

**Figure 1:**
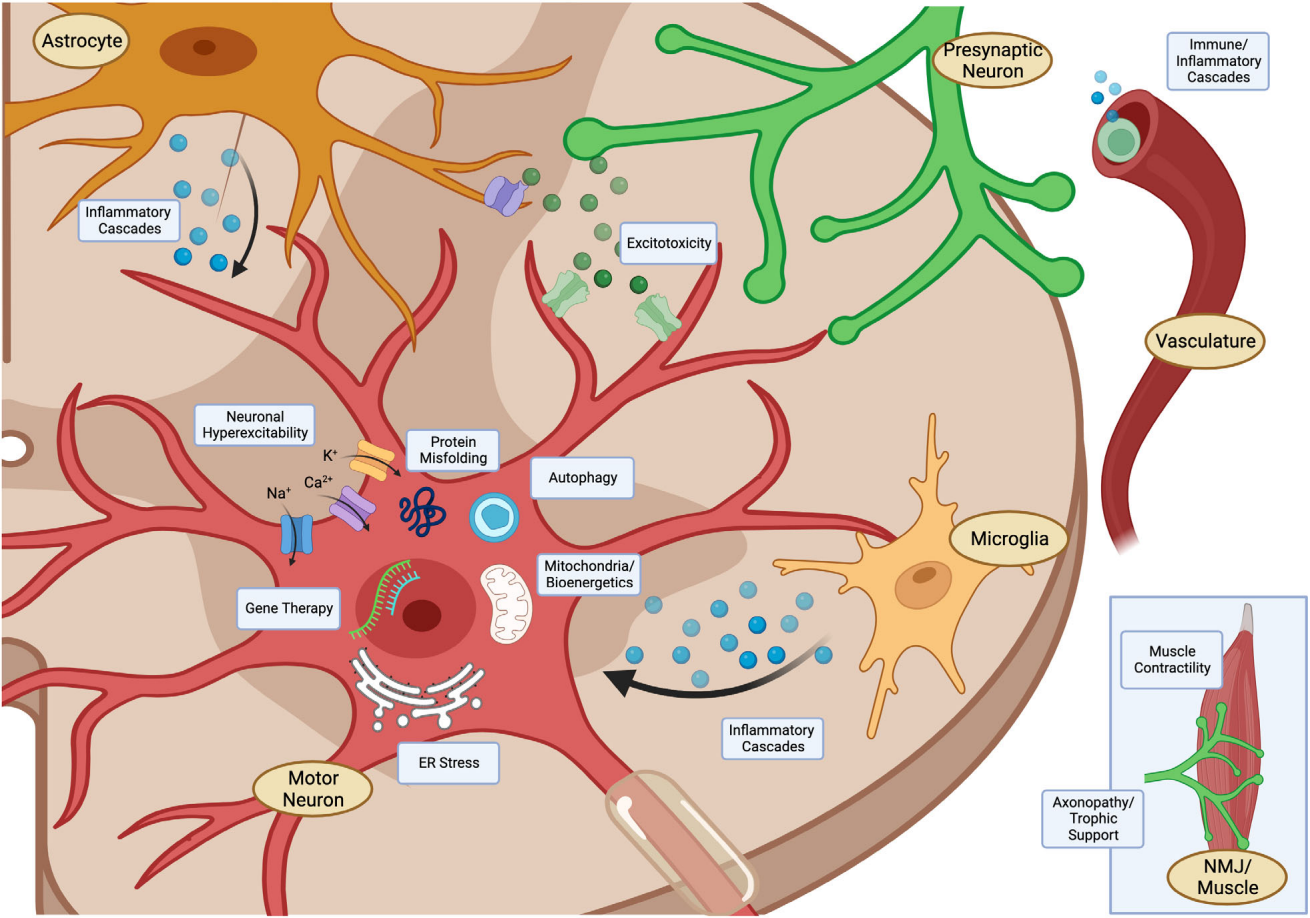
Major targets of ALS therapeutic trials. A number of ALS‐relevant pathways involving neuronal and glial cells, muscle, and systemic targets have been explored.

Along with an improved, but incomplete, understanding of ALS as a neurodegenerative disease is the evolution of more sophisticated and diverse in vitro and in vivo preclinical modeling platforms, as well as clinical trial designs. This is highlighted as we revisit proposed pathological pathways that have been major therapeutic targets for investigational compounds and whose results have been reported in the published literature (Fig. 1). It is likely that the failures of so many of these therapeutic compounds may not have occurred because of lack of efficacy but rather because of a lack of preclinical studies that would help define an appropriate disease pathway and a failure to ensure target engagement. These failures also extend to the evolution of clinical trial design in which numerous studies lacked sufficient statistical power to obtain a meaningful signal, did not stratify ALS patients who might best respond to a particular therapeutic, or lacked a biomarker that might offer clues to a therapeutic's potential efficacy (Table S1). The assertion that certain pathways are not involved in ALS pathophysiology may be the result of an inadequate or incomplete intervention that accounts for these failures. In light of this, scientists and clinicians may choose to revisit some of these therapeutic pathways and modalities reviewed here with an eye toward improving preclinical modeling, biomarker development, and the investment in more sophisticated clinical trial designs.

## Targeted Pathways in ALS Pathogenesis

### Antiglutamatergics

Because of the early observations that glutamate metabolism and glutamate neurotoxicity play a role in ALS pathogenesis, agents that targeted these pathways have been among the most well studied, both from the perspective of preclinical science as well as clinical trials in ALS.^3^, ^4^ The relative success of riluzole, whose mechanisms of action are proposed to include the inhibition of glutamate release, blockade of amino acid receptors, and inhibition of voltage‐dependent sodium channels on dendrites and cell bodies, in early ALS clinical trials resulted in the study of other agents believed to modulate glutamate excitotoxicity.^1^, ^2^ Importantly, riluzole provided a meaningful survival in ALS patients, and its efficacy, although modest, has been subsequently demonstrated over the last number of years.^5^ In particular, it seems that riluzole slows the transit between milder to more advanced stages of ALS, as confirmed more recently.^6^, ^7^ Interestingly, only two preclinical studies (an in vivo model of seizure induction and reduction and an in vitro hippocampal slice model) were referenced in the original clinical publication justifying the use of the drug in ALS.^2^, ^8^, ^9^ Confoundingly, since the original two clinical trial publications in ALS participants demonstrating the efficacy of riluzole, numerous in vitro and in vivo studies have been performed examining the mechanism and potential efficacy of riluzole in ALS models, with very mixed results regarding its neuroprotective capacity and the mechanisms of its neuroprotection.^10^, ^11^ This leaves open the question as to whether riluzole would have advanced to clinical trial for ALS today, armed with the data from these models that we now possess.

Given the modest but enduring success of riluzole in ALS, it is noteworthy that other agents modulating glutamatergic pathways have not met with success in any ALS measures. This raises the possibility that the mechanism of action and neuroprotection of riluzole may not be related to its antiglutamatergic effects alone but perhaps other modes of action, as well as its inhibition of persistent sodium current.^12^ This is highlighted by the fact that other compounds studied in ALS involved in targeting glutamate receptor subtypes like α‐amino‐3‐hydroxyl‐5‐methyl‐4‐isoxazole‐propionate (AMPA) (talampanel and topiramate) or *N*‐methyl‐d‐aspartic acid (NMDA) (dextromethorphan, memantine) have not been successful.^13^, ^14^, ^15^, ^16^ The proposed attempt to increase glutamate transporter expression, with the goal of reducing extracellular glutamate in astrocytes by using ceftriaxone, was also not successful in a large Phase 3 study.^17^ Unfortunately, in none of these trials was a biofluid or other biomarker available to help guide our understanding of target engagement or adequate inhibition of these proposed receptor subtypes.

How do we reconcile these early successes using riluzole with subsequent compounds affecting glutamatergic neurotransmission that have subsequently failed? As a group, these compounds were some of the earliest to have been studied as ALS therapies and, as such, have not benefitted from improvements in clinical trial design, including modified outcome measures and the emerging availability of biofluid biomarkers. Most of these compounds in this class underwent studies with very small sample sizes that, in the absence of dramatic clinical responses, make it difficult to assess potential signals of efficacy that would have led to larger studies. As outlined above, it may be that the efficacy of riluzole is multifactorial in its capacity to reduce excitotoxicity that subsequent compounds have been unable to reproduce. What this particular class of compounds has also lacked is a clear measure of target engagement that we are increasingly seeing in newer clinical trial designs for other compounds. Indeed, there have been no active studies of compounds directly affecting glutamatergic pathways in nearly a decade. In light of the failure of several antiglutamatergic compounds to deliver some measure of therapeutic success since riluzole, it may be challenging to expect further development of other compounds in this class without improvements in biomarker development, measures of target engagement, and/or more sensitive measures of clinical outcomes.

### Modulators of hyperexcitability

Neuronal hyperexcitability, with some interplay related to glutamate toxicity, has a long history as a postulated mechanism common to all forms of ALS. Given that there are a number of ion channel agonists and antagonists—some of which also have antiglutamatergic activity—these compounds had drawn significant attention in the late 1990s as potential mediators of disease. The broad experience with their use for other medical disorders and known side effect profiles made them attractive for study.

Compounds including gabapentin, lamotrigine, topiramate, and valproic acid not only affect glutamatergic transmission but are also ion channel blockers, reducing inward sodium current and decreasing neuronal excitability. For this reason, they have been used for disorders with well‐described patterns of neuronal hyperexcitability, including epilepsy and pain.^14^, ^18^, ^19^, ^20^

Calcium dysregulation and its subsequent downstream cascades resulting in cell death have been studied in numerous in vitro and in vivo ALS models.^21^ Therefore, reducing calcium influx via calcium channel blockade seems a reasonable approach for reducing hyperexcitability. Verapamil and nimodipine were both studied in 1996, in relatively small trials without significant changes in clinical measures, including respiratory function.^22^, ^23^ Compared with the existence of more substantial preclinical ALS‐relevant data generated today, neither of these compounds were directly studied using in vitro models of motor neuron death but relied heavily on the previously published relationships between hyperexcitability and glutamate release.

Two Phase 2 studies of mexiletine, a sodium channel blocker, failed to show any clinical or electrophysiological response in ALS, whereas another showed modest effects on measures of cortical and axonal hyperexcitability.^24^, ^25^, ^26^ Valproic acid is widely used as an antiepileptic compound because of its activity as a sodium, potassium, and calcium channel blocker, as well as its effects on γ‐aminobutyric acid (GABA) levels.^27^ Like many other compounds studied in ALS, however, it was chosen because, as a histone deacetylase (HDAC) inhibitor, it also reduces apoptosis, oxidative stress, and glutamate excitotoxicity. Despite its proposed effects on a number of potential ALS‐relevant pathways, ALS clinical measures were unaffected.^20^

As preclinical modeling in ALS has evolved, we have seen the emergence of stem cell platforms for drug discovery. This was elegantly illustrated through the selection of ezogabine from a human‐induced pluripotent stem cell motor neuron (iPSC‐MN) platform because of its capacity for reducing hyperexcitability by activating certain voltage‐gated potassium channels and improving survival of human mutant superoxide dismutase 1 (mSOD1) and C9orf72 motor neurons.^28^ Using hyperexcitability as a clinically relevant electrophysiological measure, Wainger et al.^29^ demonstrated, in an efficient 10‐week clinical trial focused on electrophysiology, that ezogabine reduced hyperexcitability by several electrophysiological measures in ALS, in particular, improving the abnormal cortical inhibitory pathway's dysfunction in patient treated with higher doses. This trial was particularly relevant because it reinvigorates potential interest in hyperexcitability as a modulator of ALS physiology; reinforces the potential preclinical predictability of using human iPSC‐MN for further development; and, because of its short duration, results in an outcome that can be pursued for further development in a later‐stage trial.^29^ Whether these preclinical and clinical electrophysiological measures predict meaningful clinical outcomes remains to be seen but provides a rational foundation for the further development of compounds that reduce neuronal hyperexcitability.

### Inflammatory cascades

Although inflammatory cascades are often considered together, it is clear from the therapeutics discussed here that the specific targets within those cascades are quite varied. Indeed, inflammatory processes of resident cells including microglia, and to some degree astrocytes, can promote a feed‐forward mechanism for the induction of an inflammatory response. However, there has also been a resurgence of the hypotheses that peripheral inflammatory processes, including those of T lymphocytes and other immune cells, may contribute to disability. These cascades are among the most varied and actively investigated ALS therapeutic targets, with a body of preclinical in vitro and in vivo modeling data to support these hypotheses.^30^

Bridging the relationship between glutamate excitotoxicity and the emerging role of inflammatory cascades in ALS, celecoxib, a cyclooxygenase‐2 (COX‐2) inhibitor of prostaglandin E2 (PGE2) synthesis, was studied for its role in reducing PGE2‐mediated release of glutamate from astroglia as well as COX‐2–induced release of cytokines.^31^ Initially, the finding that ALS patients had elevated levels of PGE2 in the cerebrospinal fluid provided the rationale for use and served as one of the early investigations of a biomarker for the disease.^32^ The study of this compound for 12 months in a double‐blinded fashion did not reach its clinical endpoint for success.^33^ However, the more interesting observation from the study was that the elevated PGE2 levels previously reported in ALS patients were not reproducible in this study. Additionally, target engagement with lowering of PGE2 levels was not obtained by celecoxib, suggesting that the drug either did not act as predicted, did not achieve adequate concentrations in the central nervous system (CNS), or that the dose/metabolism of the compound was not enough to have an effect. In retrospect, patients with elevated PGE2 levels could have been selected to be enrolled in the study, allowing for stratification of the ALS population.

NP001, a pH‐adjusted intravenous formulation of sodium chlorite, hypothesized to regulate inflammation through reduction of nuclear factor‐κB and inhibition of interleukin (IL)‐1β within monocytes/macrophages, was not found to be efficacious in clinical outcome measures, but, in an emerging effort to examine biomarkers, a subset of ALS participants who had slowing of progression (responders) were also noted to have elevated IL‐18, IL‐6, interferon gamma, and C‐reactive protein (CRP) levels when compared to nonresponders.^34^, ^35^ This spawned another Phase 2B study in which only participants with high CRP values were enrolled. Again, this compound failed to reach its primary clinical endpoint, although, as with the first study, the participants who benefitted had higher levels of CRP at enrollment.^36^ Notably, it was also discovered that CRP levels increase with age, and therefore, there may have been an overrepresentation of older ALS participants in this study.

Ibudilast is a nonselective inhibitor of phosphodiesterase that results in the reduction of leukotriene, cytokines, and other small molecules from microglia and monocytes.^37^, ^38^ A more recent preclinical study has also suggested that ibudilast may also act by inducing autophagy via mammalian target of rapamycin complex 1 (mTORC1)–transcription factor EB signaling in vitro, resulting in clearance of SOD1 and TAR DNA‐binding protein 43 (TDP‐43) aggregates.^39^ In a continued evolution for the incorporation of biomarkers, a study by Babu et al, using PBR‐28 as a PET marker of neuroinflammation combined with neurofilament light chain (NfL) as a proposed biomarker of ALS disease progression, completed an open‐label study of ibudilast that failed to show any effect on these markers.^40^ A single‐center, Phase 1b/2a study in 51 participants for 6 months, followed by a 6‐month open‐label extension, showed no change in disease progression, but a post hoc responder analysis showed that a subset of participants with a short history of ALS and progressive disease did respond.^41^ This has spawned a current Phase 2b/3 trial with those key criteria required for enrollment.^42^

Masitinib selectively inhibits the tyrosine‐kinase mast/stem cell growth factor receptor (c‐KIT) and reduces microglial activation through the blockade of colony‐stimulating factor 1 receptor.^43^, ^44^ In a study examining mSOD1 microglia in vitro accompanied by an in vivo cohort of mSOD1 rats, administration of masitinib reduced the expression of inflammatory mediators and prolonged duration of disease when administered after the onset of hindlimb paralysis.^44^ Using these data to support further development, a clinical study of this compound showed a slowing of the ALS Functional Rating Scale–revised (ALSFRS‐R) decline by approximately 20%.^45^ A confirmatory Phase 3 study is now underway (NCT 03127267).

Pioglitazone is an oral agent used for treatment of diabetes that was chosen for study in ALS because of its anti‐inflammatory properties, as well as preclinical data from three independent groups showing protection in mSOD1 mice.^46^, ^47^, ^48^, ^49^ A large Phase 2 study of the drug was stopped because of futility in extending survival in ALS participants.^50^ Unfortunately, no inflammatory biomarkers were included in the study to be able to understand whether target engagement had been obtained. The study leaned heavily on the preclinical successes in mouse models of the disease, a preclinical bias that also supported the rationale behind the failed clinical trial of minocycline, a compound believed to have anti‐inflammatory and antiapoptotic effects. For minocycline, the results were both troubling and confusing for this pathway because the compound actually appeared to accelerate disease in ALS participants.^51^

ALS pathogenesis has been associated with peripheral circulating regulatory T‐cell (Treg) levels, because their reduction promotes an increase in proinflammatory effector T cells and macrophage activation.^52^ Supported by positive results in a mSOD1 mouse model, autologous infusion of expanded Tregs in ALS patients has shown positive clinical effects.^53^ Dimethyl fumarate has been used in patients with multiple sclerosis by enhancing Treg levels in humans and reducing proinflammatory T cells.^54^ However, a randomized, placebo‐controlled, double‐blind, Phase 2 trial in ALS did not prove effective.^55^ Fingolimod is an immunomodulatory compound that antagonizes the sphingosine 1‐phosphate (S1P) receptors, blocking migration of lymphocytes from lymph organs and reducing circulating lymphocytes.^56^ Tocilizumab, a humanized monoclonal antibody antagonist of the IL‐6R, was chosen for its peripheral effects on inflammation.^57^ Both fingolimod and tocilizumab were evaluated in relatively small clinical studies that proved to be safe and well tolerated. However, of greater importance was the finding that both compounds showed evidence of target engagement allowing for future study of these compounds as mediators of peripheral as well as central inflammatory cascades.

Targeting both central and peripheral inflammatory pathways remains among the most active preclinical and clinical areas of research in ALS. Several clinical studies are underway or in planning stages, suggesting that despite some ALS failures, neuroinflammatory pathways are attractive as targeted therapeutics for neurodegeneration.^58^ Compounds targeting these pathways were historically some of the first to utilize biomarkers for patient stratification and measurements of target engagement. The more routine incorporation of biomarkers into clinical trials for ALS today owe much to the evolving attempts to incorporate neuroinflammatory biomarkers into ALS study design.

It may be that the complexity of the inflammatory response in ALS requires either combinatorial therapy or the study of additional targets. Therefore, the successes or shortcomings of results from ALS clinical trials should take into consideration that inflammatory mediators show large individual variability and do not converge on a single pathway, cell type, or even distribution within the patient. Neuroinflammatory pathways may intersect with each other, interacting with both positive and negative mechanisms for neuronal pathology. Their influences on other cell death pathways can be unpredictable, suggesting the utility of a combinatorial strategy for therapeutics.

### Trophic factors

The preclinical studies employing trophic factor administration to ALS models in vitro and in vivo have been extensive over the last three decades. The delivery of trophic factors to the CNS has remained a challenge but still provides attractive targets for investigations of viral vector delivery of these compounds to the CNS.^59^ A host of these factors have been studied in ALS participants, most notably in the late 1990s, with a number of clinical trials attempting to directly evaluate their efficacy.

The preclinical evidence that supported the advancement of ciliary neurotrophic factor (CNTF) was based on its ability to support the survival of embryonic motor neurons from stochastic cell death, prevent death of facial motor neurons from axotomy in neonatal mice, prevent death of facial motor neurons in the PMN mouse model neuronopathy, and its overall effect in the wobbler mouse of motor neuronopathy.^60^, ^61^, ^62^, ^63^, ^64^ CNTF underwent two relatively large clinical studies.^65^, ^66^ A major limitation of these studies was the development of significant side effects with peripheral administration.^67^, ^68^ Furthermore, neither of these studies showed any efficacy, which largely resulted in the abandonment of CNTF as a potential ALS therapeutic. Whether mitigating the side effect profile or a more directed delivery of the compound to the CNS without the systemic side effects could prove useful but has never been explored.

Perhaps the most thoroughly studied trophic factor was insulin‐like growth factor (IGF)‐1, which, in the first North American study, in 1997, showed an effect of slowing symptom progression.^69^ These results, using a similar clinical trial methodological design, were not reproduced in a subsequent European study.^70^ A large Phase 3 study of IGF‐1 that included a 2‐year evaluation failed to show any benefit, thus resulting in a significant shift in the field away from these targets.^71^ All three of these studies utilized subcutaneous delivery of IGF‐1, and it is unknown to what degree CNS penetration was obtained. In an attempt to bypass concerns about blood–brain barrier (BBB) penetration, a small cohort of 12 participants was administered IGF‐1 via an intrathecal route, with modest slowing of disease in this very small sample size.^72^

With preclinical data mirroring those using CNTF and with concerns about BBB penetrability, Ochs et al.^73^ performed a limited study (Phase 1/2 trial) over 12 weeks in which brain‐derived neurotrophic factor (BDNF) was infused into ALS participants intrathecally. Importantly, investigators were able to measure cerebrospinal fluid (CSF) levels of BDNF, providing some pharmacokinetics (PK) data to support the rationale behind its intrathecal (IT) administration. However, given the short timeframe of the double‐blinded portion of the study, the investigators could not make conclusions regarding efficacy of the strategy.

Vascular endothelial growth factor (VEGF) has undergone extensive preclinical studies in a number of ALS models using varied forms of delivery. One Phase 1 trial investigated the tolerability, safety, and PK of intracerebroventricularly delivered VEGF in a small group of participants, showing sustained higher levels of VEGF in CSF after treatment.^73^

Trophic factors need not be delivered systemically. Based on substantial literature about hepatocyte growth factor (HGF) and its neurotrophic effects, as well as some preclinical evidence in ALS mouse models of its role as a neuroprotectant, the hypothesis that a novel genomic complementary DNA (cDNA) hybrid human HGF injected via plasmid directly into muscle can result in the local production of HGF and subsequent maintenance of the neuromuscular junction was explored.^74^ A study incorporating injections into targeted limb muscles showed safety and tolerability.^75^ A Phase 2 study of HGF that includes sampling of muscle tissue as a biomarker has been completed (NCT04632225).

It was found that the myelin‐associated protein, Nogo‐C, is decreased in the muscle of animal models with different causes of denervation, but only in ALS patients is there a striking increase in Nogo‐A protein levels, which supports a disease‐specific mechanism causing neurite outgrowth inhibition.^76^ In a study including participants with lower motor neuron diseases, it was described that the detection of Nogo‐A in muscle biopsy samples from patients correctly diagnosed those progressing to ALS with high sensitivity and specificity.^77^ A preliminary, double‐blind trial included 40 ALS patients to receive intravenous ozanezumab (a humanized monoclonal antibody against Nogo‐A). PK results were consistent with monoclonal antibody treatments. The medication was well tolerated, but no treatment effects were observed for functional endpoints or muscle biomarkers.^78^ A larger, randomized, double‐blind, and placebo‐controlled trial with ozanezumab was negative for the primary outcome.^79^

Xaliproden, a serotonin‐1A agonist promoting release of neurotrophic factors from astrocytes, has been investigated as a trophic factor in ALS. This drug was investigated in a large, 18‐month trial including two studies: The first study, without riluzole, and a second study, with riluzole, with a total of 1210 participants. There was no influence on survival but a slightly significant positive impact on vital capacity.^80^

The potential for any future evaluation of trophic factors, including those listed above, may have to wait for more sophisticated methods of delivery to the CNS via infusion or viral vector delivery of these compounds that allow for sustained production of these factors while mitigating side effects. Furthermore, there has been the suggestion from the preclinical literature that the timing of administration of these compounds during disease may be particularly relevant. Important to the field of ALS clinical research is that trophic factors like those discussed above have also been, or are being, studied as treatments in other neurodegenerative diseases. Therefore, if improvements in delivery, CNS expression, tolerability, or efficacy are found in other patient populations, these lessons could be translated to ALS.

### Non‐cell autonomous effects in ALS and the use of stem cells as therapeutics

“Stem cell therapies” are often considered as a single therapeutic approach, but, in reality, there are numerous cell types that could be used as potential ALS therapeutics. For example, neural stem cells (NSC) with the capacity to differentiate into neurons, astrocytes, and oligodendrocytes that have been transplanted intraparenchymally are very different from mesenchymal stem cells (MSC) derived from bone marrow that have been infused intrathecally. Therefore, understanding these fundamental differences in cell types, their origin, and proposed mechanisms of action as ALS therapeutics is critical. This is especially true because the success or failure in efficacy, or a complication from stem cell therapy, could inappropriately affect the field moving forward. A complete list of the stem cell transplantation strategies for ALS is beyond the scope of this review but nicely reviewed by Je et al.^81^ Rather, this section will highlight some of the NSC and MSC delivery strategies buoyed by preclinical approaches.

Some of the earliest studies with autologous bone marrow MSC involved intraspinal transplantation into the thoracic spinal cords of ALS participants.^82^ These studies were then accompanied by a number of studies using MSC from a variety of autologous sources (bone marrow, adipose, umbilical cord).^81^ These have all been early Phase 1–2 studies focusing on safety, with small sample sizes. Many are open label and not powered to see an efficacy signal. Encouragingly, most appear to have been safe, although the results of some of these clinicaltrials.gov‐registered studies remain unpublished. The results are also complicated by the lack of biomarker data and incomplete datasets regarding cell survival and engraftment efficiency.

More recent studies of autologous bone marrow‐derived MSC that express neurotrophic factors (MSC‐NTF) deserve mention given that they were studied in a randomized, double‐blinded fashion. The first study was a Phase 2 study demonstrating safety following a single intrathecal dosing.^83^ The second study, a larger Phase 3 study utilizing repeated dosing of these cells and examining clinical efficacy, did not reach its primary endpoint.^84^ Of particular interest in these studies was the longitudinal measurements of a number of neurotrophic factors and inflammatory biomarkers that showed some patterns suggesting activity of these MSC‐NTF cells. Nonetheless, intrathecal administration of unprogrammed, autologous bone marrow‐derived MSC (including NeuroNata‐R in South Korea) is a tested option, indicating that that programmed neurotrophic factor release is not an indispensable step.^85^, ^86^

MSC seek, in theory, to supply neurotrophic factors, anti‐inflammatory molecules, and, overall, to provide a supportive milieu. NSC, however, have the capacity to differentiate into a number of neuronal and non‐neuronal cell subtypes and, in addition to the factors possessed by MSC, could engage in the recapitulation of neural networks and possibly neuronal replacement/regeneration. There are few data to suggest that the intrathecal delivery of NSC or their more mature subtypes incorporate into the brain and spinal cord in a meaningful way. Therefore, most approaches have resulted in direct intraparenchymal transplantation of these cells into the spinal cord or brain. There are a number of studies using human NSC or their derivatives in animal models of ALS (as well as other neurodegenerative diseases).^87^ However, because of the neurosurgical challenges, few have resulted in clinical trials for people with ALS. Human spinal cord‐derived NSC underwent Phase 1 and 2 studies in ALS, both of which were open label. Encouragingly, these studies showed safety of the surgical and cellular therapy, with some cells found at sites of transplantation at the time of autopsy.^88^ Combining the potential of NSC to incorporate into networks as astrocytes with an engineered overexpression of glial cell‐derived neurotrophic factor (GDNF), Baloh et al. reported the results of a Phase 1–2a trial of CNS10‐NPC‐GDNF cells into the spinal cords of ALS participants. Up to 42 months following transplantation, cells were noted at the transplant site and had GDNF expression without any significant side effects.^89^

Stem cell transplantation, particularly those incorporating NSC with the capacity to differentiate into neurons or glia, offers the potential for recapitulating neural networks and—perhaps someday—offers hope for regeneration of motor neurons. However, the current challenges for meaningful benefits from these strategies are still numerous. At the earliest preclinical stages, the transplantation and interactions of human cells with rodent cells (xenograft) for modeling therapeutic efficacy is a fundamental problem that may fall short of predicting true efficacy. Clinically, as a disease that affects the entire neuraxis, intraparenchymal delivery of these cells is limited to localized delivery. Still, proof‐of‐principle studies that examine the local efficacy of these cells can provide insights. Other hurdles both preclinically and clinically include a true understanding of cell survival following transplantation, the invasiveness of the procedures, immunosuppressive strategies, potential tumorigenicity, and others reviewed elsewhere.^90^ Whether these challenges can be overcome likely awaits advances in technological approaches for delivery and cell integration. A much more robust effort, and potentially more impactful result for ALS, is more likely to come from the utilization of stem cells, specifically human iPSC that can be differentiated into a host of ALS‐relevant cell subtypes, as platforms for understanding mechanisms of disease, biomarker development, and drug screening. Indeed, these platforms are being realized preclinically for ALS therapy development.^91^

### Gene therapies

The use of gene therapies, particularly the use of antisense oligonucleotides (ASO) acting via RNase H‐dependent cleavage of targeted RNA, has generated significant enthusiasm in the field. Importantly for ALS, the preclinical rationale behind ASO knockdown of SOD1 was strong and resulted from a number of years of methodical analyses of RNA transcript and protein levels in ALS fibroblasts, normal rats and monkeys, mSOD1^G93A^ rats and mice, and the correlation with histopathology and behavior.^92^, ^93^, ^94^

The rigorous design of the clinical trials for ASO delivery for knockdown of SOD1 established the safety of this novel strategy for intrathecal delivery to the CNS. Equally as important, however, was the incorporation of SOD1 protein measurements in the CSF as biomarkers for future study design.^95^ Several years later, a dose‐finding study of a SOD1 ASO was studied in a larger cohort of ALS participants harboring SOD1 mutations. In an evolution of design, a fast‐progressing ALS group was identified post hoc for additional analysis. The contributions of this study to the field of ALS were felt more broadly because it demonstrated a dose‐dependent reduction in SOD1 protein levels in the CSF as well as a reduction in the exploratory biomarker: NfL. Importantly, these findings seemed to correlate with a slowing of declines in functional measures, including ALSFRS‐R and slow vital capacity (SVC). These findings were particularly evident in those SOD1 ALS participants with more rapidly progressing disease.^96^ In the Phase 3 study, the stratification of SOD1 participants into fast and slow progressors was prespecified, based on the analysis of the Phase 1–2 study. Unfortunately, despite good tolerance, the primary outcome measure for efficacy, through a slowing of the ALSFRS‐R, was not realized; interestingly, however, reductions of SOD1 protein in the CSF and a reduction in NfL levels were once again noted. Results from 95 participants who entered the open‐label extension were shared. At 52 weeks, rate of ALSFRS‐R decline was significantly smaller in early treated participants compared to a delayed treated group, and a better response was also detected for SVC and handheld dynamometry.^97^ This compound, tofersen, was approved by the US Food and Drug Administration (FDA) in 2023 for treating ALS patients with SOD1 mutations. With the question of temporal administration of SOD1 ASO delivery in mind, another Phase 3 study of SOD1 ASO in asymptomatic SOD1 mutation carriers who develop early changes in biomarkers, but preceding motor deficits, is now underway (NCT04856982). Together, the preclinical data providing a strong rationale for SOD1 reduction as a therapeutic approach, combined with the rigorous and adaptive trial designs over several studies that have included patient stratification by gene mutation and progression, PK, pharmacodynamics, and biomarker data, are certain to provide a blueprint for both understanding the importance of preclinical study and also for clinical trial design.

Preclinical studies examining ASO targeting of C9orf72 hexanucleotide repeat expansion (HRE) initially leaned heavily on the use of human iPSC models in which ASOs were found to reduce C9orf72 HRE transcripts and clear intranuclear RNA foci.^98^, ^99^, ^100^ Similarly, transgenic mice with bacterial artificial chromosomes (BAC) HRE containing the *C9orf72* gene were utilized to demonstrate that ASOs targeting C9orf72 reduced HRE containing C9orf72 in the CNS and also decreased dipeptide repeat (DPR) levels.^101^, ^102^ Importantly, DPR reduction by ASOs were identified as a potential therapeutic readout with an eye toward clinical trial design. A Phase 1 trial (NCT03626012) was undertaken to deliver the ASO BIIB078 to participants with C9orf72 ALS. Although the investigational product was well tolerated, none of the primary endpoints were met. Interestingly, participants who received study drug trended toward a more rapid clinical decline. One limitation, yet to be fully evaluated, is the consideration from preclinical data that C9orf72‐targeting ASOs target only the sense strand of RNA, without targeting the antisense strand. This may lead to an incomplete reduction of RNA foci and, potentially, pathology.^103^ Another possible explanation for the resulting clinical data suggest that the loss of function related to knockdown of C9orf72 may be exacerbating symptomatology. Other clinical trials, based on their efficacy in animal models, utilizing ASOs and targeting mutations in the *FUS* ^104^ (NCT04768972) and *ATXN2* genes,^105^ (NCT04494256) are currently underway.

ASO are not the only approaches being considered for gene therapy. Preclinical study and development of RNA‐based therapeutics include small activating RNAs, adenine‐to‐inosine RNA base editing oligonucleotides, adeno‐associated virus (AAV)‐mediated gene silencing, AAV‐mediated gene correction, and AAV‐mediated gene expression activation. These approaches bring a host of additional opportunities and challenges to the therapeutic landscape.^106^ Perhaps one of the most challenging hurdles will be to address host immunogenicity to AAV administration. The translational efforts that have brought gene therapies to clinical trial have become more sophisticated with regard to their preclinical and clinical design. Utilizing a combination of human iPSC and other in vitro models, mouse and other models of disease, and the identification of potential biomarker readouts of target engagement have all spurred the advancement of these compounds to the clinic. Furthermore, these gene therapy trials have all incorporated the stratification of participants (both genetic and phenotypic), biomarker development, and advanced ALS clinical trial outcome measures. The results of these studies, even if they fail to meet primary clinical endpoints, are sure to spur conversation regarding the utility and predictability of both the preclinical and clinical models and measures.

### Modulators of oxidative stress and mitochondrial dysfunction

Inflammation and oxidative stress are related and contribute to neuronal degeneration. In particular, activation of glial cells induces oxidative stress and reactive oxygen species (ROS) resulting from multiple cellular processes and thus promoting inflammation.^107^ ROS are generated by oxidative phosphorylation, from cellular defense mechanisms and the electron transport chain complex in the mitochondria. ROS excess can cause cell death resulting from oxidative damage to nucleic acids, lipids, and proteins. Markers of oxidative stress are increased in ALS, as identified in blood, CSF, muscle, and the CNS.^108^ Antioxidant enzymes (catalase, glutathione peroxidase, and SOD), several molecules (uric acid, taurine, creatine, carotene, and flavonoids), and vitamins (C and E) can protect cells from ROS.^109^ Mitochondrial dysfunction can interfere with apoptosis, in particular concerning respiratory chain and mitochondrial permeability pore function. For this reason, it could be an interesting new target for treating ALS.

Early studies of both *N*‐acetylcysteine, acting as a reduced glutathione (GSH), and selegiline, a monoamine oxidase inhibitor with antioxidant properties, had no ALS‐relevant preclinical data, were studied in participants with ALS, and neither had any beneficial effect.^110^, ^111^

One of the earliest preclinical studies using the mSOD1 mouse as a rationale for therapeutic development included the study of vitamin E.^112^ Vitamin E (α‐tocopherol) was then tested in a study complicated by a high dropout rate, and although functional decline was smaller in the treated group, this did not reach significance.^113^ Biological evaluation of oxidative stress was performed in a subset of participants after 3 months, measuring markers of cell membrane lipid peroxidation, erythrocyte SOD activity, glutathione peroxidase (GPX) activity, and vitamin E levels. Those treated with vitamin E were observed to have higher vitamin E plasma levels, a reduction in markers of membrane lipid peroxidation, an increase in plasma GPX, but no difference in erythrocyte GPX activity nor in erythrocyte SOD activity. Using high‐dose vitamin E also failed to produce an effect on ALS survival.^114^

Creatine, as a readily available supplement, was also among the first in this category to be studied because it has antioxidant, anti‐inflammatory properties, and plays an important role in mitochondrial adenosine triphosphate production, a hallmark finding in mSOD1 mice in which the efficacy of creatine was demonstrated.^115^, ^116^, ^117^ However, in ALS participants, there was no clinical effect with either high or low doses.^118^, ^119^

Armed with both in vitro data showing its antioxidant effect as a modulator of mitochondrial activity in cell culture models and in vivo data from ALS models demonstrating a behavioral and survival effect, dexpramipexole advanced to a Phase 2 study in ALS participants, with a significant difference between groups demonstrated in a joint rank test (functional decline and mortality), thus supporting a Phase 3 trial. The large Phase 3 study failed to meet any of the endpoints, highlighting the challenges of translating smaller Phase 2 studies into Phase 3 successes. Furthermore, the absence of a biofluid biomarker further handcuffed interpretation of the results, which were initially buoyed by preclinical evidence of an effect.^120^, ^121^

Olesoxime is a small molecule with a cholesterol‐like structure, with neuroprotective properties for motor neurons in cell culture and in rodents by controlling mitochondrial permeability pore function, as well as a behavioral effect in mSOD1 mice.^122^, ^123^ A large study was subsequently undertaken without clinical benefit.^124^

Although vitamins and supplements received the most attention in the 1990s, methylcobalamin has been recently revisited. Methylcobalamin (vitamin B_12_) acts as a methyl donor for homocysteine remethylation with neuroprotective properties by decreasing the levels of neuronal homocysteine.^125^ Moreover, methylcobalamin activates extracellular signal‐regulated kinases 1 and 2 and Akt, favoring neuronal survival.^126^ Preclinical studies indicated that this compound has antiglutamatergic properties.^127^ Animal studies (wobbler mouse) indicate an effect on survival.^128^ Clinical studies in ALS have been promising regarding ventilation‐free survival in ALS participants.^129^ A large Phase 2/3 clinical trial disclosed that ultrahigh‐dose methylcobalamin (25 mg or 50 mg) was safe and well tolerated but without significant positive effects. A later post hoc analysis indicated that early affected patients (treated within 1 year of onset) with a 1‐ to 2‐point decrease in ALSFRS‐R over a 12‐week lead‐in period had a slower functional decay and longer survival.^130^, ^131^ A recent Phase 3 trial with the above features showed that, at Week 16 of the randomized period, ALSFRS‐R was 1.97 points greater in the treated group.^131^

In a demonstration of the continued evolution of preclinical ALS modeling, ropinirole was identified as a potential ALS therapeutic, through a screen of more than 1200 compounds, using cell death in hiPSC‐MN from sporadic ALS (SALS) patients as a functional readout.^132^ This compound appeared to be most active as an antioxidant but also had effects on apoptosis and protein aggregation, consistent with the multiple actions of several other ALS‐relevant compounds studied. A Phase 1/2a trial has suggested that the compound is safe and well tolerated (UMIN000034954).^133^ Whether using hiPSC‐MN from ALS patients as a preclinical tool to predict clinical success remains to be validated in a larger clinical study.

Edaravone is a free radical scavenger, capable of reducing lipid peroxides and hydroxyl radicals after neuronal insult, which was approved for treating stroke in Japan in 2001.^134^ Preclinically, this compound was studied in three ALS rodent models. In particular, work in a mSOD1 rat model showed that this drug reduced motor disability, preserved lumbar motoneurons, and resulted in a decreased 3‐nitrotyrosine (3NT)/tyrosine ratio dose dependently.^135^ In a preliminary, open clinical trial, 15 participants treated with 60 mg of edaravone intravenously showed a lesser functional decline over a 6‐month treatment period compared with a similar period before treatment. This finding was accompanied by a reduction in 3NT levels in the CSF.^136^ In a larger, 24‐week Phase 2 trial, treatment with edaravone did not prove to be effective.^137^ A Phase 3 trial with edaravone recruited a very specific Japanese population of ALS patients, with normal respiratory function, disease duration of 2 years or less, definite, or probable ALS according to the revised El Escorial criteria, scores of at least 2 points on all 12 items of ALSFRS‐R, with Grade 1 or 2 in the Japan ALS severity classification, and a decrease of 1–4 points in the ALSFRS‐R score during a 12‐week observation period before randomization. The trial revealed an effect on ALSFRS‐R decline in the treated group but without a significant impact on other functional measures.^138^ Although this trial's results permitted approval in Japan, Korea, the United States, and some other countries, it was not approved by the European Medicines Agency. Consequent population studies in the United States and Europe did not confirm a positive impact of this drug in disease progression and survival, and it could be associated with more frequent hospitalization episodes.^139^, ^140^, ^141^

The preparation of sodium phenylbutyrate (SP)/taurursodiol (TURSO), like many therapeutics discussed in this review, likely has many mechanisms of action, but an effect on endoplasmic reticulum (ER) stress and mitochondrial function is hypothesized to be the primary feature of this drug combination approved by the FDA in 2022 for the treatment of ALS. Several in vitro studies have examined these compounds separately, with an in vivo study in mSOD1 mice showing a dose‐dependent prolongation of survival and a reduction in cytochrome c release and caspase activation.^142^, ^143^, ^144^ The combination of these compounds also showed a specific transcriptional effect in SALS‐derived fibroblasts.^145^ With this proposed mechanism in mind, a Phase 2 study was conducted over 6 months showing a slowing of functional decline in the ALSFRS‐R in ALS participants taking SP/TURSO.^146^ A subsequent post hoc analysis of the data from that study also suggested a significant survival effect in ALS participants taking this compound.^147^ This compound is particularly interesting yet challenging to interpret from the standpoint of preclinical data supporting its progression to clinical trial. Although both of the compounds, studied separately, have mechanisms of action that could support their use in ALS patients, the combination of the compounds was not extensively studied preclinically in the published literature to inform about confidence in other combinatorial therapies moving forward. A large, Phase 3 study examining this compound is ongoing (NCTl05021536).

### Autophagy

The pathogenic mechanisms by which defects in autophagy pathways lead to impaired protein homeostasis in ALS remain incompletely understood. Although there is evidence that specific genetic mutations important for autophagy lead to neurodegenerative conditions including ALS, the details by which these specific mutations cause neuronal injury and a clear understanding of the neuroprotective effects of autophagy have not been determined.^148^ A few recent clinical trials have targeted aspects of autophagy, so far with largely disappointing results.

Lithium carbonate has been shown to have neuroprotective properties in vivo, in part by acting as an autophagy inducer.^149^, ^150^ Much of the original enthusiasm for lithium came from a publication in 2008 demonstrating an effect of this compound in mSOD1 mice, with a significant prolongation of survival in a relatively large cohort of ALS mice.^151^ Although specific molecular autophagic pathways were not investigated in more detail, investigators did demonstrate an increase in the number of autophagic vacuoles and the clearance of α‐synuclein, ubiquitin, and SOD1 in vitro as well as in vivo. A small study reported by the group demonstrated a remarkable effect, with all participants taking lithium alive at 15 months. However, several randomized controlled studies in ALS have not shown benefit.^152^, ^153^, ^154^, ^155^, ^156^, ^157^ The largest of these trials, the LiCALS study, lasted over 18 months but failed to show a difference in the rate of survival.^156^

Tamoxifen is another autophagy enhancer through both mTOR‐dependent and ‐independent pathways that has also been shown to reduce TDP43 protein aggregation in motor neurons and animal studies.^158^ In a small randomized controlled trial, tamoxifen was found to slow progression of ALS in a modest fashion.^159^ A comparison of 40‐ and 80‐mg daily doses of tamoxifen with 30 mg a day of creatine in a unique selective‐design Phase 2 study showed superiority of the higher dose of tamoxifen compared to the lower dose and creatine, suggesting that high‐dose tamoxifen should be compared to placebo in a future study.^160^ Other compounds currently being developed and in clinical trial with abundant preclinical evidence in several ALS models of autophagy include rapamycin (NCT03359538) and colchicine (NCT03693781).^161^, ^162^, ^163^, ^164^, ^165^, ^166^, ^167^, ^168^, ^169^

One barrier to studying this class of therapeutics in ALS is that the most effective methods for inducing autophagy are not certain. As has been previously posited, future autophagy inducers for neurodegenerative disease will require a better understanding of cell type‐specific regulatory mechanisms controlling autophagy in neuronal and nonneuronal cells in the CNS, methods to accurately measure the induction of autophagy, high‐throughput screening for autophagy induction, and alternative‐model systems such as iPSCs to test the effectiveness of these compounds.^148^

### Protein misfolding

ALS is associated with cytosolic aggregates containing specific misfolded proteins in both neuronal and glial cells that may lead to disease initiation and propagation, including SOD1 aggregates in SOD1 familial ALS and TDP43 aggregates in C9orf72 and most other forms of familial ALS, as well as SALS.^170^ Recent evidence also suggests that clinical heterogeneity in ALS may result from polyconformational misfolding of ALS‐related aggregated proteins causing prion‐like activity.^171^ A few novel inhibitors of SOD1 protein misfolding and aggregation have been studied in mSOD1 mice, most notably arimoclomol, an amplifier of heat shock proteins, which has shown the ability to rescue motor neurons from cell death in vitro and reduce protein aggregation.^172^ Much of the enthusiasm for arimoclomol was based on an improvement in mSOD1 mouse survival by a reported 22% from untreated mSOD1 mice. However, it is notable that the mSOD1 mouse cohorts included only seven mice treated with arimoclomol—a study that is considered significantly underpowered.^173^ A much larger study in 2008 showed that arimoclomol‐treated mSOD1 mice as late as 75 days of age showed a benefit when compared with untreated mSOD1 mice.^172^ Later studies using this same model showed that arimoclomol showed beneficial effects at the neuromuscular junction.^174^ A study of 38 SOD1 participants with rapidly progressing disease showed a nonsignificant trend toward a therapeutic benefit.^175^ However, a Phase 3 study of arimoclomol in a large cohort of participants with SALS did not show any benefit.^176^ Therefore, in order to determine whether arimoclomol's effect could be most appreciated in SOD1‐mediated disease—as may have been predicted by preclinical mouse modeling and early clinical studies—a larger trial specific to participants harboring SOD1 mutations would likely have to be undertaken.

Guanabenz also likely exerts its effect on protein misfolding and ER stress and was shown to be efficacious in mSOD1 mice by two separate groups.^177^, ^178^ In a Phase 2 study in which the treatment groups at three different doses were compared against historical ALS controls, the compound showed a potential benefit in the ALSFRS‐R, with fewer participants progressing to a more advanced stage of the disease. However, side effects from the drug confounded the study through a large dropout rate among individuals taking guanabenz.^179^

### Muscle

Although muscle weakness is the ultimate source of disability in ALS patients, it has largely been overlooked as a therapeutic target, likely because weakness and atrophy are thought to be bystanders of motor neuron loss rather than initiators of the process.^180^ However, given that so much is known about muscle physiology, an attempt to improve muscle contraction force at submaximal stimulation frequencies, increase power, and diminish the degree of muscle fatigue by sensitizing the troponin complex in fast‐twitch skeletal muscle fibers to calcium could improve functional motor performance in ALS despite ongoing denervation.^181^ Investigators utilized an in vitro model of human skeletal muscle to show the improvements in the response of muscle to nervous input, after which they used a rat model, which demonstrated improvements in grip strength.^182^ To address whether modulation of this target would act similarly in denervated and weakened muscle, Hwee et al. treated moderately weak mSOD1 mice with tirasemtiv and were able to demonstrate improvements in submaximal isometric force and behavioral measures in female mSOD1 mice. In a unique and informative attempt to anticipate how this compound might translate to the maintenance of respiratory function, the compound also improved both diaphragmatic contractility in these mice using an ex vivo preparation as well as pulmonary tidal capacity.^183^

With this framework in mind, several studies examined tirasemtiv, a skeletal muscle activator, for its potential in modifying functional changes related to ALS. Through a number of well‐designed studies examining dosing tolerability, interactions with riluzole, and functional measures, the compound showed some trend toward a treatment benefit in pulmonary SVC but was generally not well tolerated secondary to side effects.^184^ Therefore, in an attempt to continue to target the mechanism of action while reducing side effects, reldesemtiv, a similar compound with much fewer CNS side effects, was designed but was discontinued due to futility. Another compound with a similar, but not completely overlapping, mechanism of action, levosimendan, was also studied in a large, Phase 3 study but failed to yield any meaningful benefits.^185^

Other pathways involving muscle have attracted preclinical study that has not necessarily resulted in the study of therapeutics in ALS. Most notably, myostatin is secreted by myocytes and negatively regulates skeletal muscle growth through activin receptors.^186^ A number of companies have developed myostatin inhibitors with the intention of treating a variety of neuromuscular disorders.^187^ Preclinical studies have mostly been conducted in mSOD1 mice, with evidence of increases in muscle mass and, in some cases, strength, but not necessarily dramatic improvements in survival.^188^, ^189^, ^190^ It may be that results from other neuromuscular disorders or other preclinical models for myostatin inhibition will invigorate an interest in myostatin inhibition for ALS.

## Conclusion

With our increased understanding of ALS as a heterogeneous neurodegenerative disorder based on genetic insights, it is perhaps not surprising that most therapies for ALS as a single disease have met with failure. It is also clear that “successes” in ALS therapies have been modest at best and that better drugs should be developed. However, it would also be naïve to dismiss specific pathways or therapeutic approaches as failures based on their study in SALS over the last four decades. The efficacies of riluzole, and more recently edaravone (as a free radical scavenger) and SP/tauroursodeoxycholic acid (AMX0035) (as an antiapoptotic therapy) highlight that, although the proposed cellular pathways are not new, the efficacy of the drugs that target these pathways is relevant to current therapeutic strategies. Indeed, the mechanism of action for edaravone, approved in 2017, is hypothesized to act as a free radical scavenger.^191^ These mechanisms as mediators of ALS date back nearly 30 years. AMX0035 has been proposed as a mediator of ER stress and mitochondrial function, cascades believed to have origins in original publications from the early 1990s.^142^, ^192^, ^193^, ^194^, ^195^ How we uncover these potential successes through a combination of preclinical modeling and trial design is the challenge moving forward. If these mechanisms of action, all three distinct, are still relevant, why have other pharmacotherapies targeting these pathways failed? There are, of course, a number of possible reasons, many of which may not have been explored. It is possible that these drugs have additional mechanisms of action. This has been postulated with riluzole, for example.^11^

Drug development in ALS has been very dependent on the results of preclinical studies from the mSOD1 mouse, a model for ALS that unfortunately has inherent flaws, leading to a high failure rate in patients.^196^ The recent change to a proper pathophysiological approach will hopefully result in positive outcomes, as indicated by some recent trials.

It is noteworthy that the vast majority of both in vitro and in vivo studies have focused on spinal motor neuron pathology. There are many fewer studies examining the potential for therapeutics targeting corticospinal motor neurons (CSMN). Part of this is by virtue of the fact that rodent corticospinal tracts are anatomically unique from those of humans.^197^ Furthermore, in vitro models do not necessarily distinguish CSMN subtypes from nonspecific cortical neurons, in part because of a relative paucity of CSMN‐specific markers. However, there are preclinical studies of therapeutic compounds now beginning to address some of these shortcomings in targeting CSMN dysfunction.^198^

By the same token that we have assessed interventions in well‐established ALS‐relevant pathways, new trials, including those that target nuclear export, TDP‐43 mislocalization, and other RNA processing defects, among others, should not be judged solely on the basis of early phase studies that are upcoming.

Encouragingly, the relative successes of gene therapy for SOD1 could be attributed to the targeting of a disease‐causing mutant protein (SOD1) that was complemented by rigorous in vitro and in vivo preclinical studies in model systems overexpressing this specific mutant protein. Finally, the stratification of ALS participants with those carrying known mutations with biomarker data supporting the efficacy of the compound supports the goal of the clinical program. Conversely, the abundant preclinical data supporting ASO use in C9orf72 with similar, if not greater, in vitro and in vivo modeling using hiPSC and murine models did not predict failure of the first trial of participants in this trial. It has been hypothesized that the lack of an ASO targeting the antisense strand resulted in an incomplete knockdown of pathologically produced DPRs. Whether approaching a more complete silencing of both RNA strands will be pursued or whether other downstream targets of C9orf72 HRE will be more fruitful remains to be seen. What is evident is that the available preclinical tools for investigating these pathways is now robust.

As this review has sought to highlight, the path toward designing therapeutics targeting certain ALS pathways is littered with shortcomings that should not preclude revisiting these potential mechanisms of disease. The diversification of preclinical in vitro and in vivo tools combined with an increased understanding of genetic influences should add additional fidelity toward selecting agents that move to clinical trial. Although not a focus of this review, improvements in genotypic and phenotypic ALS patient stratification, as well as in clinical trial design, allow for more confidence in measures that suggest efficacy. Biomarker development, however, remains a challenge in bridging preclinical studies to clinical efficacy. It may be a mistake to say that a pathway is not relevant because it failed in ALS studies 20 years ago. Doing so would risk losing further development of potential drugs that might target those same pathways more effectively.

## Author Contribution

NJM JDC, and MdC wrote the manuscript.

## Conflict of Interest

NJM is a consultant to Apellis and Cytokinetics and on the scientific advisory boards of Nura Bio and Akava. He receives clinical research support from Biogen/Idec, Apellis, Helixmith, Healey Center for ALS, Calico, and Sanofi. MdC has received consulting honoraria from Cytokinetics and Kedrion. He receives clinical research support from Cytokinetics, Pfizer, Ono, and Biogen/Idec. MDW has received honoraria for serving on scientific advisory boards for Alexion, UCB‐Ra, Argenx, Biogen, Mitsubishi Tanabe Pharma, and Amylyx; consulting honoraria from Cytokinetics and CSL Behring; and speaker honoraria from Soleo Health. He also serves as a special government employee for the US Food and Drug Administration.

## Supporting information


**Table S1**. Clinical trials for targeted ALS pathways.
