# Supplementary material for: Therapeutic targeting of ALS pathways: Refocusing an incomplete picture
Source: Ann Clin Transl Neurol. 2023 Aug 28;10(11):1948–71. doi: 10.1002/acn3.51887 (PMC10647018; doi:10.1002/acn3.51887)
Supplement: Supplementary file 1 — Table S1. Clinical trials for targeted ALS pathways. [file ACN3-10-1948-s001.docx]

**Supplemental Table 1. Clinical trials for targeted ALS pathways.**

| **Pathway** | **Drug** | **Preclinical data in ALS models** | **Study phase, study design, sample size, study duration (months)** | **Target engagement/biomarker** | **Primary outcome measure and results** |
| --- | --- | --- | --- | --- | --- |
| Glutamate excitotoxicity | Riluzole^1,2^ | Effects on glutamate transport/transmission^8,11,199^ | Phase: N/A  Study design: randomized, double blind, placebo controlled  Sample size (N): 155  Duration: 12 | None reported | Outcome measure: survival  Result: improved survival in bulbar ALS and slowing of loss of muscle strength |
|  |  |  | Phase: 3  Sample Size (N): 959  Duration 18 | None reported | Outcome measure: survival  Result: improved survival but no effect on other functional measures |
|  | Gabapentin^18^ | mSOD1 mouse^112^ | Phase: 3  Sample size (N): 204  Duration: 9 | None reported | Outcome measure: rate of decline in isometric arm muscle strength  Result: no effect on primary outcome measures |
|  | Topiramate^14^ | Kainate evoked responses in cultured neurons^200^  Glut toxicity model organotypic SC culture and mSOD1 mice^201^ | Phase: N/A  Study design: randomized, double blind, placebo controlled  Sample size (N): 296  Duration: 12 | None reported | Outcome measure: ALSFRS-R and FVC  Result: patients had faster rate of decrease in arm strength and a number of side effects |
|  | Talampanel^13^ | No ALS models  Based on preclinical data from other AMPA antagonists^202–204^ | Phase: 2  Sample size (N): 59  Duration: 9 | None reported | Outcome measure: Isometric arm strength and TQNE  Result: trend toward slower decline in ALSFRS |
|  | Lamotrigine^19^ | No ALS models  Antiglutamatergic, Na, and Ca blocking effects^205–207^ | Phase: N/A  Study design: randomized, double blind, placebo controlled  Sample size (N): 30  Duration: 4 | CSF amino acids | Outcome measure: Norris Scale  Result: no effect on primary outcome measures |
|  | Dextromethorphan^15^ | NMDA antagonism using in vitro cortical and spinal motor neuron cultures^208–210^ | Phase: N/A  Study design: randomized, double blind, placebo controlled  Sample size (N): 45  Duration: 12 | None reported | Outcome measure: death, ALS Severity Scale, FVC  Result: no effect on primary outcome measures |
|  | Memantine^16^ | NMDA antagonism in mSOD1 mice^1,2^ | Phase: 2/3  Sample size (N): 63  Duration 12 | None reported | Outcome measure: ALSFRS-R  Result: no effect on primary outcome measures |
|  | Ceftriaxone^17^ | Increase in EAAT2 glutamate transporter and protection in glutamate toxicity model organotypic SC culture and mSOD1 mice^211^ | Phase: 1, 2, 3  Sample size (N): 514  Duration: >12 | None reported | Outcome measure: survival and ALSFRS-R  Result: no effect on primary outcome measures |
| Ion channel modulation | Verapamil^22^ | No ALS models  Calcium channel blocker^212^ | Phase: N/A  Study design: randomized, double blind, placebo controlled  Sample size (N): 72  Duration: 12 | None reported | Outcome measure: TQNE, FVC  Result: no effect on primary outcome measures |
|  | Nimodipine^23^ | No ALS models  Calcium channel blocker^213^ | Phase: N/A  Study design: randomized, double-blind, placebo-controlled crossover  Sample size (N): 87  Duration: 7 | None reported | Outcome measure: TQNE, FVC  Result: no effect on primary outcome measures |
|  | Mexiletine^24–26^ | Inhibition of hyperexcitability and cell death in motor neuron cell line exposed to mSOD1 astrocyte culture media^214^ | Phase: 2  Sample size (N): 60  Duration: 6 | None reported | Outcome measure: ALSFRS-R,  nerve excitability indices, death, CMAP amplitudes.  Result: no effect on electrophysiological measures of sodium current |
|  |  |  | Phase: 2  Sample size (N): 60  Duration: 3 | None reported | Outcome measures: safety and tolerability  Result: safe but not well tolerated at higher dose; dose-dependent reduction of cramp frequency and severity |
|  |  |  | Phase: 2  Sample size (N): 20  Duration: 1 | None reported | Outcome measure:  change in resting motor threshold  Results: no effect on primary outcome measure but changes consistent with inhibitor on secondary outcome measures of cortical and axonal excitability |
|  | Valproic acid^20^ | HDAC inhibition mSOD1 mice^215,216^ | Phase: N/A  Study design: randomized sequential trial  Sample size (N): 163  Duration: 16 | None reported | Outcome measure: survival and ALSFRS-R  Result: no effect on primary outcome measures |
|  | Ezogabine (retigabine)^28^ | Human ALS iPSC-MN electrophysiological assay^28^ | Phase: 2  Sample size (N): 65  Duration: 10 weeks | None reported | Outcome measure: Change in short interval intracortical inhibition  Result: positive effect on several electrophysiological measures |
| Inflammatory cascades | Celecoxib^33^ | mSOD1 mice^31^ | Phase: N/A  Study design: randomized, double blind, placebo controlled  Sample size (N): 300  Duration: 12 | No effect on CSF PGE2 | Outcome measure: change in upper extremity motor function  Result: no effect on primary outcome measures |
|  | NP001^35,36^ | No ALS models | Phase: 2  Sample size (N): 136  Duration: 6 | Subset of responders had elevated IL-18 and CRP | Outcome measure: ALSFRS-R  Result: no effect on primary outcome measures |
|  |  |  | Phase: 2B  Sample size (N): 138  Duration: 6 | No changes in PBR-28 or NfL | Outcome measure: ALSFRS-R  Result: no effect on primary outcome measures |
|  | Ibudilast^41^ | No ALS models  Models of demyelination^217–219^  Autophagy induction in NSC-34 and HEK cells, with clearing of TDP-43 and SOD1 aggregates^39^ | Phase: open label  Sample size (N): 35  Duration: 9 | NfL | Outcome measure: PBR-28 uptake measured by PET and NfL  Result: no effect on primary outcome measures |
|  | Masitinib^45^ | mSOD1 rats^44^ | Phase: 2/3  Sample size (N): 394  Duration: 12 | None reported | Outcome measure: ALSFRS-R  Result: slowing of change in the ALSFRS-R |
|  | Pioglitazone^50^ | mSOD1 mice—3 separate studies^47–49^ | Phase: 2  Sample size (N): 219  Duration: 18 months | None reported | Outcome measure: survival  Result: stopped for futility |
|  | Minocycline^51^ | mSOD1 mice—3 independent studies^220–222^ | Phase: 3  Sample size (N): 412  Duration: 9 | None reported | Outcome measure: ALSFRS-R  Result: ALSFRS-R score deterioration was faster in the minocycline group |
|  | Dimethyl Fumarate^55^ | No ALS models | Phase: 2  Sample size (N): 107  Duration: 9 |  | Outcome measure: ALSFRS-R  Result: no effect on primary outcome measures |
|  | Fingolimod^56^ | mSOD1 mice^223^  Reduction of microglial proinflammatory mediators^224^ | Phase: 2A  Sample size (N): 28  Duration: 1 | Reduction in total lymphocyte counts;  down-regulation of immune-related RNA | Outcome measure: safety  Result: safe and well tolerated |
|  | Tocilizumab^57^ | Reduction of PBMC released cytokines from ALS patients^225^ | Phase: 2  Sample size (N): 22  Duration: 4 | Reduced CRP in plasma and CSF | Outcome measure: safety and tolerability  Result: safe and well tolerated |
| Trophic Factors | CNTF^65–68^ | Embryonic motor neurons^60^  PMN mice^226^  Neonatal rat facial nerve axotomy^61^  Wobbler mice^63^ | Phase 1  Sample size (N): 4  Duration: 48 hours/cycle in 2-week cycles | CSF measurements of CNTF | Outcome measure: safety and tolerability  Result: safe and well tolerated |
|  |  |  | Phase: 1  Sample size (N): 6  Duration: 17 weeks | CNTF in CSF | Outcome measure: safety and tolerability  Result: safe and well tolerated |
|  |  |  | Phase: 3  Sample size (N): 570  Duration: 6 | None reported | Outcome measure: limb megascore  Result: no effect on primary outcome measures |
|  |  |  | Phase: 2-3  Sample size (N): 730  Duration: 9 | None reported | Outcome measure: muscle strength  Result: no effect on primary outcome measures |
|  | IGF-1^69–72^ | Glut toxicity model organotypic SC culture^227^  mSOD1 mice^228,229^ | Phase: N/A  Study design: randomized, double blind, placebo controlled  Sample size (N): 266  Duration: 9 | None reported | Outcome measure: Appel ALS rating scale  Result: slowed disease symptom progression |
|  |  |  | Phase: N/A  Study design: double blind, placebo controlled  Sample size (N): 183  Duration: 9 | None reported | Outcome Measure: Appel ALS rating scale  Result: no effect on primary outcome measures |
|  |  |  | Phase: N/A  Study design: randomized low- and high-dose IGF-1  Sample size (N): 9  Duration: 10 | None reported | Outcome measure: Norris Scale  Result: high-dose treatment slowed a decline of motor functions of the ALS patients in total Norris and limb Norris scales, but not in bulbar Norris or vital capacity |
|  |  |  | Phase: 3  Sample size (N): 330  Duration: 6 | None reported | Outcome measure: manual muscle testing, survival, ALSFRS-R  Result: no effect on primary outcome measures |
|  | BDNF^230^ | Neonatal axotomy^62^  Neonatal rat facial nerve axotomy^231^  Wobbler mouse^63^ | Phase: 1/2  Sample size (N): 25  Duration: 3 | CSF levels of r-metHuBDNF were directly related to dose | Outcome measure: safety and tolerability  Result: side effects limiting upper dose |
|  | VEGF^73^ | mSOD1 mice^232,233^ | Phase: 1  Sample size (N): 18  Duration: 3 | CSF VEGF | Outcome measure: safety and tolerability  Result: safe |
|  | HGF^75^ | SOD1 mice^74^ | Phase: 1/2  Sample size:18  Duration: 9 | Transient changes in copy number VM202 plasmid DNA, HGF protein | Outcome measure: safety and tolerability  Result: safe |
|  | Ozanezumab^79^ | Up-regulation of Nogo-A expression in muscle of SOD1 mice model and in patients with ALS^234^ | Phase: 2  Sample size (N): 303  Duration: 12 | No evidence of pharmacological response with ozanezumab treatment on protein or RNA biomarkers | Outcome measure: ALSFRS-R and survival (joint-rank analysis)  Result: no effect on primary outcome |
|  | Xaliproden^80^ | PMN mice^235^ | Phase: 3  Study 1, sample size (N): 867—xaliproden vs. placebo 18 months—EFC2941  Study 2, sample size (N): 1,210—xaliproden + riluzole vs. xaliproden + placebo 18 months—EFC 1923 | None reported | Outcome measure: time to death, tracheostomy or permanent  assisted ventilation, and time to VC < 50%.  Result: no effect on primary outcome measures. Trend toward an effect on VC |
| Stem cells | MSC-NSC^83^ | No ALS models but evidence of neuroprotection in other models of neurodegeneration^236^ | Phase: 2  Sample size (N): 48  Duration: 6 | Effects on neurotrophic factors, inflammatory biomarkers, and microRNA expression levels | Outcome measure: safety and tolerability  Result: Safe. In a prespecified rapid progressor subgroup, rate of disease progression was improved at early time points |
|  |  |  | Phase: 3  Sample size (N): 196  Duration: 7 | Changes in neuroinflammatory and neurodegenerative biomarkers, including MCP-1 and NfL | Outcome Measure: ALSFRS-R  Result: no effect on primary outcome |
|  | BM-MSC^86^ |  | Phase: 2  Sample size (N): 64  Duration: 6 | Changes in cytokine profiles | Outcome Measure: ALSFRS-R  Result: changes in ALSFRS-R were reduced in MSC group compared to controls |
|  |  |  |  |  |  |
|  | Human SC-derived NSC-HSSC^88,237^ | mSOD1 rats^238^ | Phase: 1  Sample size (N): 18  Duration: 48 | None reported | Outcome measure: safety and tolerability  Result: safe and well tolerated. |
|  |  |  | Phase: 2  Sample size (N): 15  Duration: to death | None reported | Outcome measure: safety and tolerability  Result: safe and overall well tolerated |
|  | Human NSC^239^ | mSOD1 rats^240^ | Phase: 1  Sample size (N): 18  Duration: 36 | None reported | Outcome measure: safety and tolerability  Result: safe |
|  | Human NSC-GDNF^89^ | A number of ALS rodent models^241–244^ | Phase: 1-2a  Sample size (N): 18  Duration: 42 | Transplanted cell survival in SC demonstrated; GDNF expression in transplanted regions | Outcome measure: safety  Result: safe |
| Gene therapies | SOD1 ASO (ISIS 333611)^95^ | Reduction of SOD1 in fibroblasts, rats, and monkeys^92^  mSOD1 mice and rats^93^  Reduction in SOD1 protein in CSF^245^ | Phase 1  Sample size (N): 22  Duration: 1 | Drug CSF concentration | Outcome measure: safety, tolerability, and PK  Result: no dose-limiting toxic effects or any safety or tolerability concerns |
|  | SOD1 ASO (tofersen)^96,97^ |  | Phase: 1/ 2  Sample size (N): 48  Duration: 3 | Reduction in SOD1 protein levels following tofersen delivery | Outcome measure: safety and PK  Result: safe, although CSF pleocytosis in treatment group |
|  |  |  | Phase: 3  Sample size (N): 72  Duration: 7 | Reduction in SOD1 protein levels and NfL following tofersen delivery | Outcome measure: ALSFRS-R  Result: no effect on primary outcome measure |
| Antioxidants and modulators of mitochondrial dysfunction | *N*-Acetyl Cysteine^110^ | No ALS models | Phase: N/A  Study design: Randomized, double blind, placebo controlled  Sample size: 110  Duration: 12 | None reported | Outcome measure: survival  Result: no effect on primary outcome measures |
|  | Selegiline^111^ | No ALS models | Phase: N/A  Study design: Randomized, double blind, placebo controlled  Sample size: 133  Duration: 6 | None reported | Outcome measure: Appel ALS Score  Result: no effect on primary outcome measures |
|  | Vitamin E (low and high dose)^113,114^ | Delayed onset and slowed progression in mSOD1 mice^112^ | Phase: N/A  Study design: Randomized, double blind, placebo controlled  Sample size: 289  Duration: 12 | Increases in plasma vitamin E, with reduced markers of lipid peroxidation | Outcome measure: survival  Result: no effect on primary outcome measures |
|  |  |  | Phase: N/A  Study design: randomized, double blind, placebo controlled  Sample size: 160  Duration: 18 months | None reported | Outcome measure: survival  Result: no effect on primary outcome measures |
|  | Creatine^118,119^ | mSOD1 mice either alone or in combination with other compounds^115–117^ | Phase: N/A  Study design: Randomized, placebo- controlled, sequential trial  Sample size: 175  Duration: 12 months | None reported | Outcome measure: survival  Result: no effect on primary outcome measures |
|  |  |  | Phase: N/A  Study design: Randomized, double blind, placebo controlled  Sample size: 104  Duration: 6 months | Urinary creatinine | Outcome measure: maximum voluntary isometric contraction  Result: no effect on primary outcome measures |
|  | Dexpramipexole^120,121^ | Inhibition of cell death in vitro and slowing of behavioral decline in mSOD1 mice | Phase: 2  Sample size:v102  Duration: 6 months | None reported | Outcome measure: ALSFRS-R  Result: reduction in slope of ALSFRS-R decline |
|  |  |  | Phase: 3  Sample size: 943  Duration: 12-18 months | None reported | Outcome measure: CAFS Score  Result: no effect on primary outcome measures |
|  | Olesoxime^124^ | Rat embryonic MN protection in vitro and mSOD1 mice^123^ | Phase: 2/3  Sample size: 512  Duration: 18 months | None reported | Outcome Measure: Survival  Result: No effect on primary outcome measures |
|  | Methylcobalamin^131^ | Methylcobalamin reduced mSOD1 mouse MN cell death^249^ | Phase: 3  Sample size: 130  Duration: 16 weeks | Plasma homocysteine | Outcome measure: ALSFRS-R  Result: slowed the decline in ALSFRS-R by 45% |
|  | Edaravone^136–138^ | mSOD1 mice^250^  mSOD1 (H46R) rats^135^  Wobbler mice^251^ | Phase: 2  Sample size (N): 20  Duration: 6 | Reduction in CSF NT3 | Outcome measure: ALSFRS-R  Result: slowing of decline of ALSFRS-R |
|  |  |  | Phase: 3  Sample size (N): 204  Duration: 6 | None reported | Outcome measure: ALSFRS-R  Result: no effect on primary outcome measures |
|  |  |  | Phase: 3  Sample size (N): 137  Duration: 6 | None reported | Outcome measure: ALSFRS-R  Result: slowing of decline of ALSFRS-R |
|  | Sodium Phenylbutyrate /TUDCA^146,147^ | mSOD1 mice (NaPB)^144^  ALS fibroblasts^145^  Neuroblastoma cells (NaPB)^142^  Isolated mitochondria (TUDCA)^143^ | Phase: 2  Sample size: 137  Duration: 6 | None reported | Outcome measure: ALSFRS-R  Result: slowed the decline in ALSFRS-R |
| Autophagy | Lithium^152–156^ | mSOD1 mice^151^ | Phase: 2  Sample size (N): 171  Duration: 15 | None reported | Outcome measure: Survival or severe loss of autonomy  Result: no effect on primary outcome measures  Poorly tolerated |
|  |  |  | Phase: 2  Sample Size (N): 88  Duration: up to 13 | None reported | Outcome measure: time to a decrease of at least six points  on the ALSFRS-R or death  Result: no effect on primary outcome measures |
|  |  |  | Phase: 2  Sample size (N): 107  Duration: 13 | None reported | Outcome measure: change in ALSFRS-R  Result: no effect on primary outcome measures |
|  |  |  | Phase: 2b  Sample size (N): 133  Duration: 16 | None reported | Outcome measure: survival  Result: no effect on primary outcome measures |
|  |  |  | Phase: 3  Sample size (N): 214  Duration: 18 | None reported | Outcome measure: survival  Result: no effect on primary outcome measures |
|  | Lithium + valproate^157^ |  | Phase: 2  Sample size (N): 42  Duration: 18 | None reported | Outcome measure: change in ALSFRS-R and survival  Result: slowing of rate of decline of ALSFRS-R and improved survival |
|  | Tamoxifen^159^ | FTLD-U mouse model with TDP-43 proteinopathy^158^ | Phase: 2  Sample size (N): 18  Duration: 12 | None reported | Outcome measure: time to death or mechanical ventilation  Result: modest slowing of rate of decline of ALSFRS-R at 6 months |
|  | Tamoxifen vs. creatine^160^ |  | Phase: 2  Sample size (N): 60  Duration: 38 | None reported | Outcome measure: change in ALSFRS-R  Result: slowing of rate of decline of ALSFRS-R and improved survival for high-dose tamoxifen |
| Protein quality control (misfolding) | Arimoclomol^175,252^ | mSOD1 mice treated presymptomatically^173^  mSOD1 mice treated at symptom onset^253^ | Phase: 2  Sample size (N): 84  Duration: 3 | Arimoclomol in CSF increased with dose | Outcome measure: safety, tolerability, and PK  Result: safe and well tolerated  No clinical effect |
|  |  |  | Phase: 2/3 (adaptive design)  Sample size (N): 38  Duration: 12 | None reported | Outcome measure: safety and tolerability and survival  Result: safe and well tolerated  Suggestion of possible clinical effect on survival, change in ALSFRS-R, and CAFS |
|  | Guanabenz^179^ | mSOD1 mice^177,178^ | Phase: 2  Sample size (N): 201  Duration: 6 | No change in NfL | Outcome measure: change in ALS-MITOS  Result: slowed disease progression |
| Muscle | Tirasemtiv^184,254,255^ | Activation of fast skeletal muscle troponin^181,182^  mSOD1 mice^183^ | Phase: double blind, placebo controlled in 3 parts  Sample size (N): 49  Duration: 22 days | None reported | Outcome measure: safety and tolerability  Result: safe and tolerable with reduced dose of riluzole |
|  |  |  | Phase: 2b  Sample size (N): 711  Duration: 3 | None reported | Outcome measure: ALSFRS-R  Result: no effect on primary outcome |
|  |  |  | Phase: 3  Sample size (N): 744  Duration: 6 | None reported | Outcome measure: SVC  Result: no effect on decline in SVC |
|  | Levosimendan^185,256^ | No ALS models | Phase: 2  Sample size (N): 66  Duration: 14 days | None reported | Outcome measure: SVC  Result: no effect on SVC |
|  |  |  | Phase: 3  Sample size (N): 496  Duration: 12 | None reported | Outcome measure: supine SVC at 12 weeks and survival (CAFS) at 48 weeks  Result: no effect on outcome measures |
|  | Reldesemtiv^257^ | No ALS models | Phase: 2  Sample size (N): 458  Duration: 12 | None reported | Outcome measure: SVC  Results: no effect on primary outcome measure but trends to efficacy in several measures |
| ALS, amyotrophic lateral sclerosis; ALSFRS-R, ALS Functional Rating Scale–Revised; AMPA, α-amino-3-hydroxy-5-methyl-4-isoxazolepropionic acid; ASO, antisense oligonucleotide; BDNF, brain-derived neurotrophic factor; CAFS, combined assessment of function and survival; CMAP, compound muscle action potential; CNTF, ciliary neurotrophic factor; CRP, C-reactive protein; CSF, cerebrospinal fluid; EAAT2, excitatory amino acid transporter-2; FTLD-U, frontotemporal lobar degeneration with ubiquitin-positive inclusions; FVC, forced vital capacity (pulmonary); GDNF, glial cell–derived neurotrophic factor; HDAC, histone deacetylase; HEK, human embryonic kidney; HGF, hepatocyte growth factor; HSSC, human skeletal stem cells; IGF-1, insulin-like growth factor 1; IL, interleukin; iPSC-MN, induced pluripotent stem cell–motor neurons; MCP-1, monocyte chemoattractant protein-1; MiToS, Milano-Torino staging; MN, motor neurons; MSC, mesenchymal stem cells; mSOD1, mutant superoxide dismutase 1; NaPB, sodium phenylbutyrate; NfL, neurofilament light chain; NMDA, N-methyl-D-aspartic acid; NSC, neural stem cells; PBMC, peripheral blood mononuclear cells; PBR, peripheral benzodiazepine receptor; PET, positron emission tomography; PK, pharmacokinetics; r-metHuBDNF, recombinant methionyl human brain-derived neurotrophic factor; SC, spinal cord; SOD1, superoxide dismutase 1; SVC, slow vital capacity (pulmonary); TDP-43, TAR DNA-binding protein 43; TQNE, Tufts Quantitative Neuromuscular Exam; TUDCA, tauroursodeoxycholic acid; VC, vital capacity; VEGF, vascular endothelial growth factor. | | | | | |
